# Control of *Escherichia coli* in Fresh-Cut Mixed Vegetables Using a Combination of Bacteriophage and Carvacrol

**DOI:** 10.3390/antibiotics12111579

**Published:** 2023-10-30

**Authors:** Maryanne Kuek, Sarah K. McLean, Enzo A. Palombo

**Affiliations:** Department of Chemistry and Biotechnology, School of Science, Computing and Engineering Technologies, Swinburne University of Technology, Hawthorn, VIC 3122, Australia; smclean@swin.edu.au (S.K.M.); epalombo@swin.edu.au (E.A.P.)

**Keywords:** bacteriophage, phage biocontrol, carvacrol, fresh-cut mixed vegetable, antimicrobial, combined treatment

## Abstract

The continual emergence of antibiotic-resistant bacteria and the slow development of new antibiotics has driven the resurgent interest in the potential application of bacteriophages as antimicrobial agents in different medical and industrial sectors. In the present study, the potential of combining phage biocontrol and a natural plant compound (carvacrol) in controlling *Escherichia coli* on fresh-cut mixed vegetable was evaluated. Four coliphages, designated *Escherichia* phage SUT_E420, *Escherichia* phage SUT_E520, *Escherichia* phage SUT_E1520 and *Escherichia* phage SUT_E1620, were isolated from raw sewage. Biological characterization revealed that all four phages had a latent period of 20–30 min and a burst size ranging from 116 plaque-forming units (PFU)/colony forming units (CFU) to 441 PFU/CFU. The phages effectively inhibited the growth of respective host bacteria in vitro, especially when used at a high multiplicity of infection (MOI). Based on transmission electron microscopy analysis, all phages were classified as tailed phages in the class of *Caudoviricetes*. Additionally, next generation sequencing indicated that none of the selected coliphages contained genes encoding virulence or antimicrobial resistance factors, highlighting the suitability of isolated phages as biocontrol agents. When a phage cocktail (~10^9^ PFU/mL) was applied alone onto fresh-cut mixed vegetables artificially contaminated with *E. coli*, no bacteria were recovered from treated samples on Day 0, followed by a gradual increase in the *E. coli* population after 24 h of incubation at 8 °C. On the other hand, no significant differences (*p* < 0.05) were observed between treated and non-treated samples in terms of *E. coli* viable counts when carvacrol at the minimum inhibitory concentration (MIC) of 6.25 μL/mL was applied alone. When a phage cocktail at an MOI of ~1000 and MIC carvacrol were applied in combination, no *E. coli* were recovered from treated samples on Day 0 and 1, followed by a slight increase in the *E. coli* population to approximately 1.2–1.3 log CFU/mL after 48 h of incubation at 8 °C. However, total elimination of *E. coli* was observed in samples treated with a phage cocktail at a higher MOI of ~2000 and carvacrol at MIC, with a reduction of approximately 4 log CFU/mL observed at the end of Day 3. The results obtained in this study highlight the potential of combined treatment involving phage biocontrol and carvacrol as a new alternative method to reduce *E. coli* contamination in minimally processed ready-to-eat foods.

## 1. Introduction

Foodborne illnesses cause significant morbidity and mortality worldwide, especially in young, elderly and immunocompromised individuals. In Australia alone, it is estimated that there are 5.4 million foodborne gastroenteritis incidents each year which result in approximately 15,000 hospitalizations and 80 deaths [1]. Food products can be easily contaminated with bacteria throughout the food supply chain, leading to foodborne illness. To ensure global food demands are met in a world where the population is constantly growing, antibiotics are often used in intensive food production systems for the purpose of treating diseases and improving the health of livestock. However, the overuse of antibiotics leads to the development of resistant bacteria, thereby reducing the effectiveness of the drugs while indirectly increasing the cost of treatment and rate of mortality in clinical settings. The presence of resistance genes in food products poses a serious risk as these genes can be easily spread across the human population once they enter the food chain [2,3,4,5]. With the continuous increase in antibiotic-resistant bacteria and the scarcity of new antibiotics, the World Health Organization (WHO) has predicted that by 2050 the world will be facing the same threats as in the pre-penicillin era [6,7,8]. As a result, bacteriophage have been widely studied in recent decades in the hope of finding alternatives to combat bacterial infections and to solve healthcare issues arising from antibiotic-resistant bacteria [3]. Nonetheless, some studies have reported the limited efficacy of phage treatment due to the emergence of phage-resistant mutants [9,10,11].

Other than phages, essential oils (EOs) extracted from various parts of plants have also been used as natural antibacterial agents to control bacteria growth. The chemical components found in EOs play an important role in the defense mechanism of plants against pests, bacteria and fungi in the natural environment [12,13,14] and have been exploited for medicinal applications. For example, oregano (*Origanum vulgare*), a medicinal plant from the family of *Lamiaceae*, is commonly found in the Mediterranean and Asia [12]. Different parts of the plant are used as a food flavor enhancer and conventionally used as a traditional medicine to treat diseases like asthma, cramping, diarrhea and indigestion [15,16]. The extracted aromatic oil known as oregano essential oil (OEO) is composed of various volatile components such as terpenes, aldehydes, ketones and ethers with reported strong anti-inflammatory, antibacterial and antioxidant activities [15,17]. While OEO consists of many different chemical components, carvacrol (5-isopropyl-2-methylphenol) is identified as the main component and has been proven to possess antibacterial properties [15]. The concept of combining bacteriophages with essential oils as antimicrobial methods was first proposed by Viazis and colleagues in 2011; however, not many studies with such combinations have been conducted since then [18].

In recent times, the fresh-cut produce industry has undergone rapid growth due to an increase in consumer demands for healthy, fresh and convenient food products [19]. The nutrient-rich inner surface of fresh food products exposed after peeling, slicing and trimming promotes the growth of microorganisms. Since fresh produce is often consumed without further processing, this increases the risk of developing foodborne illness upon consumption. Hence, the present study aimed to assess the effectiveness of combined treatment involving newly isolated coliphages and carvacrol against *Escherichia coli* on fresh-cut mixed vegetables (iceberg lettuce, carrot and purple cabbage) in condition simulated to reflect “real world” settings. To the best of our knowledge, this is the first study conducted using a combined treatment of bacteriophages and carvacrol to control *E. coli* populations on fresh-cut mixed vegetables. The majority of the studies conducted to date have focused primarily on one type of fresh produce at a time [20,21,22,23,24,25,26,27,28].

## 2. Results

### 2.1. Isolated Coliphages and Their Host Range

Screening of sewage samples collected from the Eastern (ETP) and Western Treatment Plants (WTP) in Victoria, Australia, yielded 17 coliphages with distinct plaque morphology against different primary host strains (*E. coli* O157:H7, *E. coli* K12 and *E. coli* ATCC 25922). Distinct plaques were selected, purified and propagated to obtain high-titer phage stocks. The isolated phages were designated *Escherichia* phage SUT_E120 to *Escherichia* phage SUT_E1720, accordingly. The isolated phages were then tested against 17 *E. coli* strains and nine *Salmonella* strains (Table 1) to determine their respective host ranges. SUT_E1620 was observed with the broadest host spectrum, being able to infect nine *E. coli* strains and four *Salmonella* strains. On the other hand, SUT_E420 and SUT_E520 infected five tested *E. coli* strains, while SUT_E1520 could only infect three out of the seventeen *E. coli* strains tested. When confirmatory plaque assays were conducted, the plaques observed when plated with susceptible strains appeared slightly turbid and are generally smaller in size. The selected phages were also reported with a low (EOP ≥ 0.001) to high (EOP ≥ 0.5) efficiency of plating (EOP) against different susceptible strains (Supplementary Material; Table S1). Four phages (SUT_E420, SUT_E520, SUT_E1520 and SUT_E1620) were selected and subjected to further biological characterization as they showed a wider host range compared to others.

### 2.2. Morphological Analysis of Coliphages under TEM

**Figure 1:**
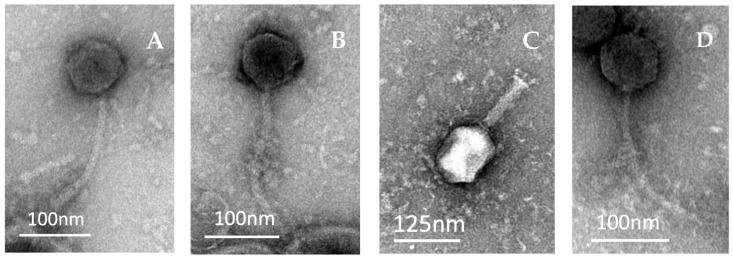
Electron micrograph showing the morphology of (**A**) SUT_E420; (**B**) SUT_E520; (**C**) SUT_E1520 and (**D**) SUT_E1620.

The morphologies of SUT_E420, SUT_E520, SUT_E1520 and SUT_E1620 observed under transmission electron microscopy (TEM) are shown in Figure 1. All four phages were classified into the order of *Caudovirales* (tailed phages). SUT_E420, SUT_E520 and SUT_E1620 (Figure 1A,B,D) were observed with a long, flexible tail, with an isometric head, suggesting that these phages most likely belong to the family of *Siphoviridae*. On the other hand, SUT_E1520 has a typical morphology of a *Myoviridae* phage, having a long contractile tail with a base plate and short tail spikes (Figure 1C). The length and the width of the head and the tail of phages were measured and calculated by averaging at least five measurements obtained via Image J (FIJI) software (version 2.15.0) (Table 2).

### 2.3. One-Step Growth Curve of Coliphages

**Figure 2:**
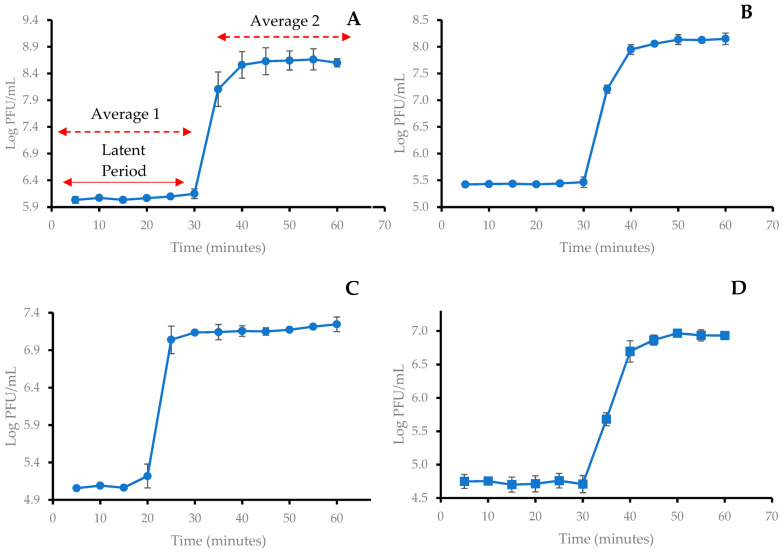
One-step growth curve of (**A**) SUT_E420; (**B**) SUT_E520; (**C**) SUT_E1520; (**D**) SUT_E1620. Points plotted represent the average values of 3 independent replicates; error bars represent the standard deviations (SD) of the mean values.

One-step growth curves were carried out to determine the latent period and burst size of the selected phages. The mean values of three independent experiments were used to plot the one-step growth curves (Figure 2), and the latent period and burst size were calculated (Table 3). All the phages were observed with very similar latent periods ranging from 20 and 30 min. The burst sizes of SUT_E420 and SUT_E520 were higher, having 350 and 441 PFU/CFU, respectively, while SUT_E1520 and SUT_E1620 were observed to have lower burst sizes of 116 and 140 PFU/CFU, respectively.

### 2.4. Bacterial Growth Kinetics after Phage Treatment at Different Multiplicities of Infection (MOI)

**Figure 3:**
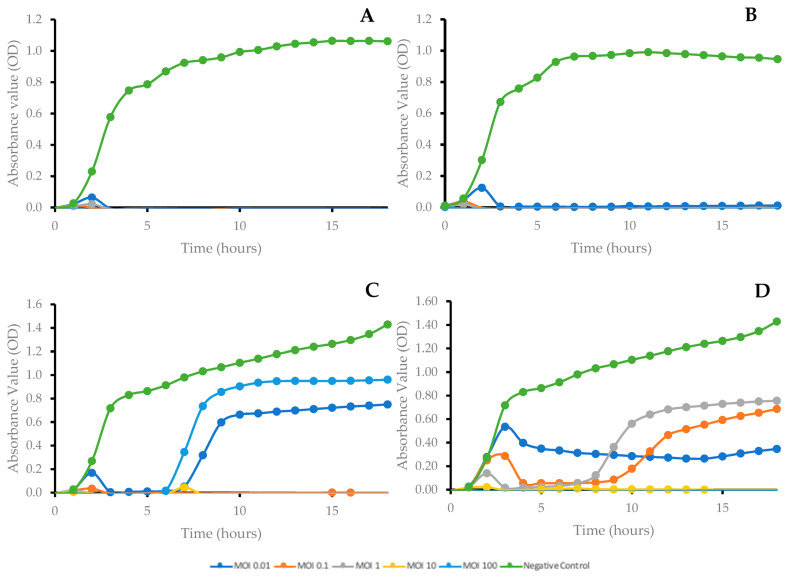
Growth curves of host bacteria after phage treatment at different MOI (0.01, 0.1, 1, 10 and 100). (**A**) SUT_E420 + *E. coli* O157:H7; (**B**) SUT_E520 + *E. coli* O157:H7; (**C**) SUT_E1520 + *E. coli* K12; (**D**) SUT_E1620 + *E. coli* K12. Points plotted represent the average values of 3 replicates.

Differences were observed in the absorbance readings between phage-treated samples and controls, despite the MOI used. The absorbance readings obtained shown that both SUT_E420 and SUT_E520 were able to inhibit the growth of their host strain (*E. coli* O157:H7) for 18 h regardless of the MOI applied. The optical density of each well remained close to the level of blank, indicating that the bacteria were lysed before reaching detectable levels (Figure 3A,B). As for SUT_E1520 (Figure 3C), the optical density of the cultures was maintained close to the level of blank for the first 6 h; however, the outgrowth of host bacteria (*E. coli* K12) after the sixth hour was observed in wells with phage added at a MOI of 0.01 and 100. Meanwhile, the application of SUT_E1620 at a MOI of 10 and 100 resulted in complete inhibition of host bacteria (*E. coli* K12) throughout the 18 h. Although the addition of SUT_E1620 at MOI of 0.1 and 1 delayed the start of host bacterial growth, the outgrowth of host bacteria still occurred after the ninth and seventh hour, respectively (Figure 3D).

### 2.5. Genomic Analysis of Coliphages via Next Generation Sequencing (NGS)

The complete genomes of all four phages were sequenced and deposited in the GenBank database with the accession numbers OQ990039, OQ990040, OQ885479 and OQ939939 for SUT_E420, SUT_E520, SUT_E1520 and SUT_E1620, respectively. Genomic analysis revealed that all four phages have linear double stranded DNA (dsDNA) with a total length ranging from 102,434 bp to 113,861 bp. No known virulence or antimicrobial resistance genes were identified in any of the phages, confirming the safety for use as biocontrol agents.

When compared with sequenced phages in the NCBI database, the BLASTn results showed that SUT_E420, SUT_E520 and SUT_E1520 have the greatest similarity hits with Escherichia phage T5_ev212 (accession no. LR597659.1) and Escherichia phage EC148 (accession no. ON185585.1), with a percentage match of approximately 99%. On the other hand, SUT_E1620 showed the greatest percentage match (98.03%) with Phage vB_SabS_Sds2 (accession no. MW357609.1), a novel virulent phage isolated from the feces of donkeys in China [29]. The following section will only provide a detailed description of the ORFs annotated from the SUT_E1620 genome and their functionality, since it was observed to have the most notable variations compared to other sequenced phages.

**Figure 4:**
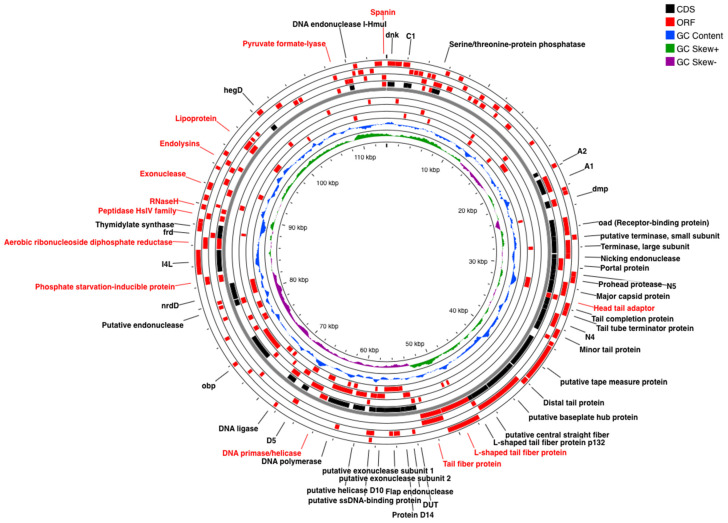
Genomic map of SUT_E1620. Hypothetical proteins with unknown function are not shown. The figure was visualized and annotated using Proskee software. The rings from inside out represent GC skew (green and purple), GC content (blue), CDS (black) and ORF (red) annotated by Prokka Annotator Version 1.0.0. The annotated ORFs labelled in red were predicted by the NCBI ORF finder.

SUT_E1620 was revealed to possess linear dsDNA with 113,861 bp with an average coverage of 589×. In total, 25 tRNAs and 200 genes were predicted, from which 175 functional proteins were annotated, including 98 hypothetical proteins with unknown function. The functional genes responsible for encoding phage structural proteins and cell lysis proteins, as well as phage DNA replication and packaging proteins were annotated and labeled in Figure 4. Proteins associated with phage DNA replication consisted of a DNA primase, DNA polymerase, DNA ligase, putative helicase D10 and putative ssDNA-binding protein. On the other hand, phage structural proteins such as a major capsid protein, head tail adaptor, tail tube terminator protein, tail completion protein, minor tail protein, distal tail protein, putative tape measure protein and L-shaped tail fiber protein were annotated as well, together with proteins that assist in regulating phage metabolism activities (serine/threonine-protein phosphatase, endonuclease, transcriptional regulator, NAD-dependent protein deacylase, phosphate starvation-induced protein, dihydrofolate reductase, A1 and A2 protein). Spanin protein that helps in releasing phage progenies by completing the host cell lysis process via the breaking of the host cell outer membrane was annotated in combination with endolysins and holin (C1 protein) [30,31]. Such observations suggested that SUT_E1620 was likely using a holin-endolysin cell lysis system to target host bacteria.

**Figure 5:**
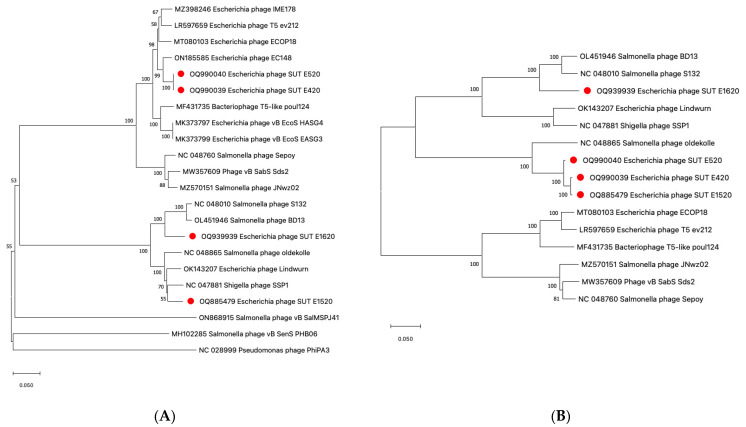
Phylogenetic tree constructed based on (**A**) large terminase subunit; (**B**) receptor-binding proteins (RBPs) using MegaX software. The phylogenetic tree was generated using the neighbor-joining method with 1000 bootstrap replicates. Numbers at nodes represent the bootstrap percentage. Phages isolated in this study are indicated by red dots.

The phylogenetic analysis was conducted on the large terminase subunits (Figure 5A) as it is identified as one of the most conserved parts of phage genomes [29]. The analysis conducted revealed that SUT_E420 and SUT_E520 are closely related, both sharing the same branch in the same cluster with a 100 bootstrap percentage. The result indicated that the sequences used to encode large terminase subunit are most likely identical among the two phages. SUT_E1520 and SUT_E1620 appeared in a different cluster, sharing the same branch with other closely related reference phages. A phylogenetic tree constructed based on sequences encoding *oad* genes (receptor-binding protein RBP; UniProt Identifier P23207) revealed that SUT_E420 and SUT_E1520 were the most closely related, sharing the same branch, while SUT_E520 shared the same cluster on a separate branch (Figure 5B). A possible reason for this observation could be the ability of SUT_E520 to infect some of the Salmonella strains tested (Section 2.1), suggesting a slightly different RBP when compared to SUT_E420 and SUT_E1520. Interestingly, SUT_E1620 formed a separate cluster with other Salmonella phages on the tree and appeared to be distantly related to the other isolated coliphages. None of the closely related reference phages shared the same branch with SUT_E1620, highlighting its uniqueness and possible consideration as a novel phage.

### 2.6. Minimum Inhibitory Concentration (MIC) of Carvacrol In Vitro

Based on the agar dilution assay, 6.25 μL/mL was identified as the minimum inhibitory concentration (MIC) of carvacrol against streptomycin-resistant *E. coli* (*E. coli* StrR), the strain used in biocontrol studies described below.

### 2.7. Sensitivity of Coliphages towards Carvacrol

**Figure 6:**
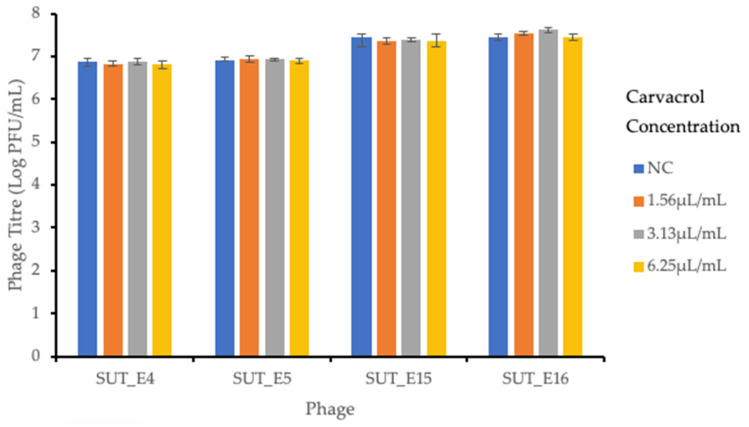
Coliphages sensitivity to carvacrol. The sensitivity of coliphages against different concentrations of carvacrol was evaluated after 24 h incubation at 8 °C. NC; negative control. Error bars indicate the standard deviation of three independent experiments.

The sensitivity of phages towards carvacrol was tested to ensure the lytic activity and viability of phages was not affected by the addition of carvacrol. The results shown in Figure 6 indicate that no significant (*p* > 0.05) differences were observed in the phage titers of samples with and without added carvacrol. This result showed that there was a minimal effect on the viability of isolated coliphages after overnight incubation with carvacrol at 8 °C.

### 2.8. Combined Treatment of Coliphages and Carvacrol against E. coli StrR on Fresh-Cut Mixed Vegetables

**Figure 7:**
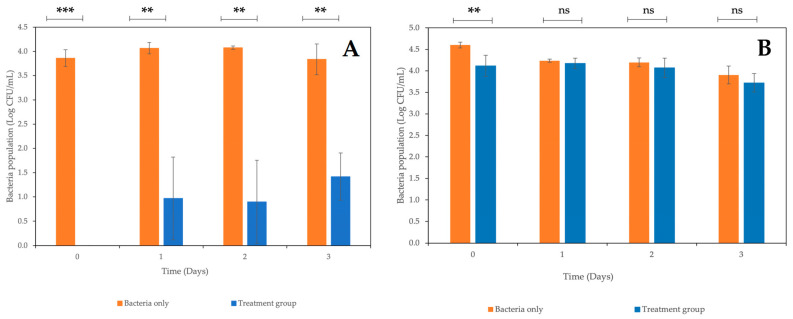
Effect of (**A**) coliphage cocktail; (**B**) carvacrol at MIC in reducing *E. coli* StrR on fresh-cut mixed vegetables stored at 8 °C over 4 days. Data plotted represent the mean values of 3 biological replicates; error bars indicate the standard deviation of the average values. *** means a significant difference of *p* < 0.001; ** means a significant difference of *p* < 0.05; ns means not significant (*p* > 0.05).

Fresh-cut mixed vegetables were artificially contaminated with *E. coli* StrR and treated with a phage cocktail consisting of the four newly isolated coliphages, carvacrol or a combination of the two antibacterial agents. The effects of a coliphage cocktail and carvacrol against *E. coli* StrR when applied alone are shown in Figure 7A,B. When the coliphage cocktail was applied alone, no *E. coli* were recovered from phage-treated samples on Day 0, with a reduction of approximately 3.8 log CFU/mL being observed (Figure 7A), after which the population of *E. coli* recovered from treated samples and increased to approximately 0.9–1.4 log CFU/mL. Nevertheless, the level of recoverable bacteria from treated samples was significantly lower (*p* < 0.05) than those recovered from non-treated samples across the experimental period, with a reduction of 2.4 log CFU/mL still being observed at the end of Day 3.

As for samples treated with carvacrol at MIC (6.25μL/mL), the *E. coli* population recovered from treated samples was comparable with that recovered from the non-treated samples across the experimental period (Figure 7B). No significant differences (*p* > 0.05) were observed between treated and non-treated samples in terms of *E. coli* viable counts starting from Day 1, while a significant reduction (*p* < 0.05) in *E. coli* population was observed on Day 0 with a reduction of approximately 0.49 log CFU/mL.

**Figure 8:**
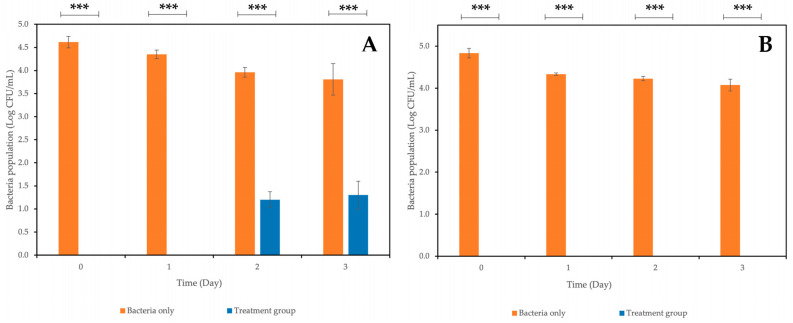
Effect of combined treatment involving coliphage cocktail and carvacrol (MIC) at (**A**) MOI of ~1000; (**B**) MOI of ~2000 in reducing *E. coli* StrR on fresh-cut mixed vegetables stored at 8 °C over 4 days. The data plotted represent the mean values of 3 biological replicates, and error bars indicate the standard deviation of the average values. *** means a significant difference of *p* < 0.001.

On the other hand, the combined application of a coliphage cocktail (~10^9^ PFU/mL) at an MOI of ~1000 and carvacrol at MIC resulted in no recoverable *E. coli* in treated samples on Day 0 and 1 (Figure 8A), followed by a slight increase in the *E. coli* population to approximately 1.2 log and 1.3 log CFU/mL on Day 2 and 3, respectively, in treated samples. When a phage cocktail was applied at a higher MOI of ~2000 in combination with carvacrol at MIC, no *E. coli* were recovered from all treated samples across the experimental period (Figure 8B). The *E. coli* population recovered from the non-treated samples was approximately 4 log CFU/mL greater than that reported in the treated samples. Overall, the *E. coli* population recovered from treated samples was significantly lower (*p* < 0.001) than those reported in the non-treated samples when a phage cocktail was applied in combination with carvacrol.

These combined treatments resulted in a greater reduction in *E. coli* populations than when individual treatments were applied, suggesting possible synergistic effects between isolated phages and carvacrol. The results obtained also revealed that the magnitude of the combined treatment was MOI-dependent, as treatment with a higher MOI resulted in a greater reduction in the *E. coli* population.

## 3. Discussion and Conclusions

Considering the rapid evolution of antibiotic-resistant bacteria and the slow development of new antibiotics, phages have received widespread attention by utilizing their bactericidal properties as alternatives to antibiotics [7,8]. In the present study, four coliphages, designated SUT_E420, SUT_E520, SUT_E1520 and SUT_E1620, were examined. A host-range analysis showed that SUT_E1620 has the widest host spectrum, and, along with SUT_E520, was able to infect *Salmonella* strains. When concentrated phage stock was spotted onto lawns of susceptible bacteria, clear plaques were observed. This could be due to the occurrence of ‘lysis from without’ activity, as large numbers of viral particles applied can cause numerous holes on the bacterial cell membrane leading to cell lysis even before phage replication [32,33]. The turbid plaques observed could also be due to partial lysis of bacterial cells as phage binds less effectively to those susceptible strains compared to their primary host strain [34].

A short latent period and large burst size are ideal for phages used for biocontrol purposes, as this indicates that a large number of virions can be produced in a shorter time frame, and hence enable more effective lysis of the target host strains [35,36]. The latent periods of selected coliphages (Table 3) fell within the normal expected range for a tailed phage. The results obtained were comparable to those reported in Ateba & Akindolire [35], where a latent period of 12–30 min was reported. Interestingly, the burst sizes calculated for SUT_E420 and SUT_E520 were much larger than commonly reported burst sizes, which typically range from 50 to 200 PFU/CFU [37,38,39,40]. The growth kinetic curves of bacteria (Figure 3) showed that the ability of phage to inhibit the growth of host bacteria varied with the type of phage applied. Some of the phages were more effective at higher phage–bacterial ratios (higher MOI), while others were more effective at a lower MOI [20,25,36,38]. In the study conducted by Wójcicki et al. [31], the complete elimination of host bacteria was achieved when the four highest MOI (1.0, 10, 100 and 1000) were used. On the other hand, an optimum MOI of 0.1 was reported for Phage vB_VpS_BA3 and Phage vB_VpS_CA8 in the study conducted by Yang et al. [41]. It is interesting how samples treated with SUT_E1520 at an MOI of 100 had a greater outgrowth of bacteria (Figure 3C) compared to samples treated with a lower MOI. A possible reason for this observation could be the formation of bacterial mutants which replaced the phage-sensitive population and led to the re-growth of bacterial cells [42]. The emergence of insensitive mutants was also predicted in samples treated with a lower MOI. However, no attempt was made in the present study to further assess whether the resistance developed was permanent or temporary. Regardless of the optimum MOI determined, a phage cocktail at a higher concentration was administered onto artificially contaminated fresh-cut mixed vegetables in Section 2.8, as previous authors observed that the application of a higher concentration of phage can increase the probability of direct contact between bacteria and phage [36].

Analysis conducted in Image J (FIJI) software showed that the sizes of the heads and tails of SUT_E420, SUT_E520 and SUT_E1620 were within the normal range reported for *Siphoviridae* phages, which typically have a 50–80 nm wide isometric head and a flexible tail with a length ranging from 100 to 168 nm long and 4 to 9 nm wide [37,38,43]. However, the abolishment of morphology-based families (*Siphoviridae*, *Myoviridae* and *Podoviridae*) along with the order *Caudovirales* by ICTV in their latest taxonomy release prevents the classification of newly isolated phages merely based on the morphology. This highlights the importance and significance of performing comprehensive genomic analysis for newly isolated phages to allow classification according to their genomic characteristics. The phylogenetic and genomic comparative analysis conducted has enabled the classification of all isolated coliphages into the class *Caudoviricetes*, which is the newly proposed class by ICTV to group tailed phages with dsDNA and icosahedral capsids [44]. The BLASTn search showed that SUT_E420, SUT_E520 and SUT_E1520 had the greatest nucleotide similarity with *Escherichia* phage T5_ev212 (~99%) and *Escherichia* phage EC148 (~99%), while SUT_E1620 shared the greatest similarity with Phage vB_SabS_Sds2 (~98%). According to Li et al. [25], a minimum genomic similarity of 95% is required to meet the species classification standard of ICTV for bacterial and archaeal viruses. Therefore, based on this guideline, SUT_E420, SUT_E520 and SUT_E1520 could be classified as members of the genus *Tequintavirus* of the family *Demerecviridae*, while SUT_E1620 most likely belongs to the genus *Epseptimavirus* of the family *Demerecviridae*.

The complete genome analysis revealed that all four phages have linear double-stranded DNA (dsDNA) with a total length ranging from 102,434 bp to 113,861 bp. Most of the CDS and ORFs annotated in the coliphage genomes are homologues and their putative functions can be identified based on the findings of previous studies. The A1 and A2 proteins annotated from SUT_E1520 and SUT_E1620 genomes are purported to support phage infection by inhibiting the synthesis of host genes and protein while at the same time destroying the host DNA [29]. The ORF encoding the L-shaped tail fiber protein was predicted in all coliphages. This structural protein is associated with bacterial lipopolysaccharide (LPS) O-antigen specific binding, which helps to increase the adsorption rate of phages onto bacterial strains with O-antigen receptors [29]. Although the receptor-antigen bindings of the O-antigen are claimed to be less specific than phage central fibers and their corresponding receptors, the ability of phages to rapidly bind to bacteria O-antigen receptors allows them to infect their hosts more effectively, especially when host numbers are frequently less than those of phages in the natural environment [29]. The annotation of this protein suggested that all isolated coliphages could be classified as O-antigen targeting phages. Additionally, the dihydrofolate reductase and thymidylate synthase encoded were predicted to work together to reduce 7,8-dihydrofolate to tetrahydrofolate which helps in the conversion of dUMP to dTMP by acting as a co-factor in enzymatic reactions [25,45]. On the other hand, HNH endonucleases which help to regulate phage transcription were believed to play a crucial role in phage evolution by allowing the mobilization of genes. As for endolysin, holin (C1 protein) and spanin, they are proteins associated with the cell lysis module. These proteins were predicted in all coliphages and are believed to play an important role in the host cells’ lysis system by breaking the host peptidoglycan and perforating the cell membrane to enable the release of virion progeny [25,46]. Furthermore, approximately 23–25 tRNAs were annotated in all coliphage genomes. The presence of tRNAs is believed to affect the phage dependency towards its host bacteria. This is because the distribution of tRNA is highly correlated with codon usage which indirectly facilitates phage replication by corresponding to codons used by phage rather than those used by the hosts [47,48]. The absence of genes encoding lysogenic markers such as integrase, recombinase, repressors and excisionase also indicate the virulent and lytic nature of selected coliphages. Moreover, the absence of genes encoding virulence and antibiotic-resistance factors in all coliphages confirmed the safety of using these phages as biocontrol agents.

Among the cases of foodborne diseases reported, outbreaks caused by the consumption of contaminated fresh produce such as fruits and vegetables account for one-third of the major outbreaks [49,50]. According to the Centers for Disease Control (CDC), 59,736 foodborne outbreaks were reported from 2015 to 2021 in the United States, out of which 19,203 cases were related to the consumption of contaminated fresh produce, which resulted in 11,344 hospitalization and 703 deaths [51]. The application of phages as biocontrol agents in the fresh produce industry is well documented, with studies conducted on lettuce, spinach, cantaloupe, apple, broccoli and tomato in recent decades [20,22,23,27,32,38,52]. However, none of the previous studies looked at the potential of phage biocontrol on fresh-cut mixed vegetables. A key and novel feature of the current study was that the vegetables were used without prior decontamination. This enabled the applied agents to be evaluated under real-world conditions among a background of normal microbiota. The results showed that when a coliphage cocktail (~10^9^ PFU/mL) was applied alone, a reduction of approximately 3.8 log CFU/mL in the *E. coli* viable count was observed in treated samples on Day 0. Despite the fact that *E. coli* regrowth occurred after 24 h, the population recovered from treated samples remained significantly (*p* < 0.05) lower than the non-treated samples. Interestingly, no significant differences were observed between treated and non-treated samples in terms of *E. coli* viable counts when carvacrol at MIC was applied. The *p* value (*p* = 0.186) obtained from two-way ANOVA shows that the change in the *E. coli* concentration over time is not statistically different between treated and non-treated samples. Similar observations were made in previous studies and authors claimed that the MIC value determined in model laboratory systems might be much lower than the MIC value needed to inhibit bacteria growth in real food systems, as internal interactions with food components such as fats, proteins and carbohydrates can indirectly reduce the antibacterial activity of the chemical component on food products [27,53,54]. However, increasing the applied concentration of carvacrol is not feasible in this setting as high levels of carvacrol may cause changes in the organoleptic properties of fresh produce which are often consumed without further processing [55]. Hence, treatment involving a phage cocktail and carvacrol was proposed in this study in the expectation that the combination of both treatments would enhance the overall bactericidal activity. This combination is of particular interest as Viazis et al. [18] and Di Pasqua et al. [56] claimed that EOs can access the periplasm of cells without disturbing the outer membrane or destroying the intracellular ATP pools. Such a mechanism allows phages to attach more readily to the bacterial membrane, thus enabling phage to infect host bacteria with greater efficiency.

Indeed, the total elimination of *E. coli* was achieved with a phage cocktail at an MOI of ~2000 and MIC carvacrol. Similar observations were made in a study conducted by Chang et al. [57], as the application of carvacrol and endolysin (LysSA97) encoded by phage SA9 was able to reduce the population of *Staphylococcus aureus* in milk samples more effectively than when individual treatments of endolysin and carvacrol were applied. The authors observed an approximately 0.8 and 1.0 log reduction in viable count after individual treatment of endolysin LysSA97 (376 nM) and carvacrol (3.33 mM), respectively, while a reduction of ~4.5 log CFU/mL was observed when treatments were applied in combination. In another study conducted by Moon et al. [58], the potential of carvacrol (1.6% *w*/*v*) and a commercial phage product (SALMONELEX) in controlling *Salmonella* on chicken pieces was investigated. The results showed that a significantly greater reduction (~1.9–2.0 log CFU/g) in *Salmonella* was observed during the sequential application of carvacrol and SALMONELEX. In contrast, individual SALMONELEX (10^8^ PFU/mL) or carvacrol (1.6% *w*/*v*) treatment only resulted in a reduction of approximately 0.9 log and 1.6 log CFU/g, respectively. All these previous studies showed that phages or components of phages (e.g., endolysin) and carvacrol can act in a complementary manner to further reduce bacterial numbers when applied in combination. However, the present study did not assess whether the addition of carvacrol would affect the palatability of treated vegetables. Future studies involving sensory evaluation of fresh produce after treatment would be necessary to ensure the proposed combined strategy is feasible in real-world applications and will not result in a residual oil taste in fresh produce. Despite the settings of the current studies demonstrating the potential of phage biocontrol when applied in the presence of background microbiota, additional studies are recommended to further explore and refine the application of phage–carvacrol biocontrol, as only one temperature parameter was used for testing. Future studies can investigate the effect of fluctuating incubation temperatures that simulate real-world conditions on the total amount of bacteria that can be recovered. Nevertheless, the classification of EOs as Generally Recognized as Safe (GRAS) and the approval of several phage products by the European Food Safety Authority (EFSA), Food and Drug Administration (FDA) and Food Standard Australia New Zealand (FSANZ) to be used commercially on food products in recent years indicate the potential of this combined strategy as a new alternative for antibiotics [55,59,60].

## 4. Materials and Methods

### 4.1. Characterization of Newly Isolated Coliphages

#### 4.1.1. Host Bacteria Strains and Culture Conditions

A total of 17 *E. coli* strains and 9 *Salmonella* strains (Table 1) were used to determine the host range of isolated phages, from which *E. coli* O157:H7, *E. coli* K12 and *E. coli* ATC 25922 were selected as the primary isolation strains. All strains were part of a culture collection at Swinburne University of Technology. The bacteria were cultured in BD Bacto^TM^ Tryptic Soy Broth (TSB, Thermo Fisher Scientific, Melbourne, Australia) at 37 °C with shaking at 190 rpm. Overnight cultures were used for all experiments, except in the primary isolation of phages where 4 h exponential phase cultures were used.

#### 4.1.2. Isolation of Coliphages and Transmission Electron Microscope (TEM) Analysis

Phages were isolated from sewage samples collected from the Eastern and Western Treatment Plants in Melbourne, Australia. Briefly, 15 mL of sewage water was aliquoted into a 50 mL Falcon tube and centrifuged for 20 min at 4000× *g*. The supernatant was filtered through a 0.22 μm filter and stored at 4 °C until further use. The presence of lytic phages was confirmed by plaque assay with *E. coli* O157:H7 (EDL 933), *E. coli* K12 and *E. coli* ATC 25922 as the primary isolation strains. Distinct plaques observed were picked using sterilized 1000 μL pipette tips and immersed in 3 mL salt magnesium (SM) buffer for purification. The same procedures were repeated thrice to prepare pure phage lysate. Then, high-titer phage stocks were prepared using the plate propagation method previously described by Carey-Smith et al. [37].

Five microliters of concentrated phage stock (~10^10^ PFU/mL) was transferred to respective mesh copper grids with a formvar carbon support film (Microscopy Solution Pty Ltd., Melbourne, Australia). The samples were then negatively stained twice with 5 μL of 2% uranyl acetate and blotted with Whatman filter paper to remove excess stain. The grids were stored at room temperature before viewing under the Jeol JEM 2100 electron microscope. The width and length of the head and tails of the phages were analyzed using Image J (FIJI) software (version 2.15.0) (National Institute of Health (NIH)).

#### 4.1.3. Host Range of Phages

Spot testing was used to determine the host range of isolated phages. Seven microliters of concentrated phage lysate (10^9^ PFU/mL) were spotted onto bacterial lawns prepared with fresh overnight culture and left to dry with the aid of laminar flow cabinet. Plates were then incubated overnight at 37 °C.

Following incubation, phage(s) which formed clear lysis zones were subjected to plaque assay to the determine the efficiency of plating (EOP) of susceptible *E. coli* strains with reference to the primary host strain. The EOP was calculated using the formula stated in Huang et al. [38]:

$$\mathrm{Efficiency}\;\mathrm{of}\;\mathrm{plating}\;\left(\mathrm{EOP}\right)=\frac{\mathrm{Average}\;\mathrm{phage}\;\mathrm{titre}\;\mathrm{on}\;\mathrm{susceptible}\;\mathrm{strain}\;\left(\frac{\mathrm{PFU}}{\mathrm{mL}}\right)}{\mathrm{Average}\;\mathrm{phage}\;\mathrm{titre}\;\mathrm{on}\;\mathrm{primary}\;\mathrm{host}\;\mathrm{strain}\;\left(\frac{\mathrm{PFU}}{\mathrm{mL}}\right)}$$

#### 4.1.4. One-Step Growth Curve of Phages

The one-step growth curve experiment was carried out based on the methodology described by Kropinski et al. [61] with slight modification. Briefly, the bacterial host was grown to exponential phase and adjusted to OD_600_ of 0.5. Then, 100 μL of diluted phage lysate (~10^6^–10^7^ PFU/mL) was added to the absorption flask containing 900 μL of exponential phase (OD_600_ of 0.5) bacteria. The phage–bacterial suspension was then incubated for 5 min at 37 °C in a shaker incubator with continuous stirring to allow the absorption of phage to its host bacteria. After incubation, the phage–bacterial suspension was centrifuged at 10,000× *g* for 1 min at 4 °C to remove free phages. Then, 100 μL of phage-bacterial suspension was transferred from the absorption flask to Flask A, followed by the transfer of 1 mL suspension from Flask A to Flask B, then from Flask B to Flask C. All the flasks were then placed in the shaker incubator at 37 °C and incubated for 1 h with sampling at 5 min intervals. At various time points, 80 μL of phage–bacterial suspension was removed from the appropriate flask (Flask A, B or C) and the phage titer was determined via plaque assay. The phage latent period and burst size (refer to Figure 2) were calculated using the formula stated by Kropinski et al. [61] with slight modification.

$$\mathrm{Burst}\;\mathrm{size}\;\left(\frac{\mathrm{PFU}}{\mathrm{CFU}}\right)=\frac{\mathrm{Average}\;\mathrm{phage}\;\mathrm{titre}\;\mathrm{after}\;\mathrm{burst}}{\mathrm{Average}\;\mathrm{phage}\;\mathrm{titre}\;\mathrm{before}\;\mathrm{burst}}\;\;$$

#### 4.1.5. Growth Kinetics of Bacterial Hosts following Phage Treatment at Different MOI

The optimum MOI of each selected phage was determined based on the methodology described by Chen et al. [62] and Yang et al. [41]. Briefly, exponential phase (OD_600_ 0.5) bacteria were serially diluted to approximately 10^6^ CFU/mL and 150 µL transferred to each well in sterile 96-well plates. Phage stock lysates were then serially diluted from ~10^9^–10^5^ PFU/mL and 15 μL of each concentration was transferred to the appropriate well to give a MOI of 0.01, 0.1, 1, 10 and 100. Sterile broth, phage lysate and exponential phase bacteria were used as blank, positive and negative controls, respectively. The plates were incubated at 37 °C for 18 h in a FLUOstar^®^ Omega Plate Reader (BMG LabTech, Ortenberg, Germany) and the absorbance (OD_600_) of each well measured at 30 min intervals. Readings from 3 wells were collected for each of the tested MOI and the mean values were used to plot graphs.

#### 4.1.6. Genomic Analysis of Phages Using Next Generation Sequencing (NGS) Technology

Phage DNA was extracted using a commercial kit purchased from Norgen Biotek (Phage DNA Isolation Kit; Product #46800, 46850). Based on the manufacturer’s instructions, extracted DNA was used as the input for Econoflex library preparation followed by DNA sequencing on an NGS MiniSeq Mid-Output kit with 150 bp paired end reads. Reads were assembled using SPAdes version 3.15.3 to construct contig sequences. Phages with multiple contigs were assembled using SnapGene 7.0 to obtain a single nucleotide sequence and later annotated with Prokka (version 1.14.6). Annotated open reading frames (ORFs) were confirmed with NCBI ORF finder (https://www.ncbi.nlm.nih.gov/orffinder/, accessed on 30 April 2023), and phage complete assemblies were then visualized using Proskee software—https://proksee.ca/ (accessed on 30 April 2023) [63]. The Comprehensive Antibiotic Resistance Database (CARD) Resistance Gene Identifier (RGI) (version 1.2.0) and VirulenceFinder 2.0 (version 2.0.3) (https://cge.food.dtu.dk/services/VirulenceFinder/, accessed on 30 April 2023) were used to analyze the antimicrobial resistance and virulence factors, respectively. The phage genomes were deposited into the GenBank database and assigned unique accession numbers.

Phylogenetic analysis of the phage large terminase subunit and receptor binding protein was carried out using MegaX software (version 10.2.6). The neighbor-joining statistical method with 1000 bootstrap replicates was used to construct phylogenetic trees.

### 4.2. Analysis of Carvacrol

#### 4.2.1. Preparation and Analysis of Carvacrol

Carvacrol (>99%) was purchased from Sigma-Aldrich Pty Ltd. (Sydney, Australia) and emulsified in 2% Tween-20 in TSB, followed by 90 s of homogenization by vortexing at high speed to achieve a starting concentration of 200 µL/mL. The emulsified carvacrol was then serially diluted in TSB with 2% Tween-20 to achieve different concentrations ranging from 100 µL/mL to 0.05 µL/mL. Carvacrol stocks were prepared fresh on the day of the experiment.

#### 4.2.2. Antimicrobial Activity of Carvacrol

The minimum inhibitory concentration (MIC) of carvacrol was determined using the agar dilution method described by Huang et al. [38]. Carvacrol was first emulsified and prepared as stated above. Then, 5 µL of different concentrations of carvacrol (100 µL/mL to 0.05 µL/mL) was spotted onto tryptic soy agar (TSA) spread with overnight bacteria suspension and further incubated at 37 °C for 24 h. The MIC was interpreted as the last concentration where no visible bacteria growth (clear lysis zone) was observed.

#### 4.2.3. Susceptibility of Phages to Carvacrol

Phage stability towards different concentration of carvacrol were tested using the method described previously by Ni et al. [14]. Phage lysates were diluted to ~10^7^ PFU/mL and incubated with carvacrol at a concentration of ¼ MIC (1.56 µL/mL), ½ MIC (3.13 µL/mL) and MIC (6.25 µL/mL) for 24 h at 8 °C. The phage titer after incubation was determined via plaque assay.

### 4.3. Application of Phage Cocktail and Carvacrol to Fresh-Cut Mixed Vegetables

#### 4.3.1. Bacterial Inoculum

Streptomycin-resistant *E. coli* K12 (*E. coli* StrR) was used to artificially contaminate fresh-cut mixed vegetables. The strain was selected for streptomycin resistance based on the methods described by Viazis et al. [18]. Briefly, the original isolate underwent serial passaging on TSA supplemented with increasing concentrations of streptomycin (Sigma-Aldrich, St. Louis, MO, USA). At least five serial passages were carried out before the strain was considered as streptomycin resistant. The final product was able to grow in the presence of 300 µg/mL streptomycin. This allowed selection and identification of *E. coli* against a background of normal food microbiota in biocontrol studies.

An overnight bacteria culture was prepared for the food biocontrol experiments. The cells were harvested the following day by centrifugation and resuspended in phosphate buffered saline (PBS). Resuspended bacteria were then adjusted to OD_600_ of 0.5 and further diluted to 10^6^ CFU/mL. The bacterial suspension was used to artificially contaminate the fresh-cut mixed vegetables.

#### 4.3.2. Preparation of Phage Cocktail and Carvacrol

Each phage was diluted to the desired titer with SM buffer and then an equal volume of each phage was combined to prepare a phage cocktail. Fresh cocktails were prepared on the day of the experiment.

Carvacrol was emulsified and prepared as stated in Section 4.2.1. The different combinations of phage cocktail and carvacrol were prepared by diluting carvacrol in phage cocktails prepared beforehand to give a solution that contained approximately MIC (6.25 µL/mL) of carvacrol in the final product.

#### 4.3.3. Antimicrobial Properties of Phage Cocktail and Carvacrol on Fresh-Cut Mixed Vegetables

Fresh-cut mixed vegetables consisting of iceberg lettuce, carrot and purple cabbage were purchased from a local supermarket immediately before each experiment. The plastic packaging was first wiped with 70% ethanol to remove external microorganisms.

To ensure a consistent starting quantity, mixed vegetable samples were weighed into corresponding containers. Then, approximately 6 log CFU/mL of fresh overnight culture of *E. coli* StrR was delivered onto mixed vegetables with a small fingertip sprayer that delivers 100 μL per spray. Samples were then air-dried for 45 min in a biosafety cabinet to enable bacterial attachment before spraying the treatment group with phage cocktail, carvacrol or various combinations of phage–carvacrol mixtures. PBS was used as the negative control. After 10 min, 10 g of mixed vegetables (treated or controls) was weighed, packed individually into plastic bags and sealed with a heat sealer. Each bag was filled with nitrogen gas (N_2_) to mimic the real-life scenario of a fresh produce processing facility and stored for 4 days at 8 °C (±1–2 °C) to simulate mild temperature abuse conditions that may occur during transport or storage. At the appropriate time point, samples were removed from the incubator, 50 mL of PBS was added and the sample homogenized. Aliquots containing recovered bacteria were then serially diluted and spread plated onto TSA containing 300 μg/mL of streptomycin. *E. coli* were enumerated after overnight incubation at 37 °C.

### 4.4. Statistical Analysis

Three replicates were prepared for all experiments in this study. The data presented are expressed as the mean value ± standard deviation. The sensitivity of phages towards different concentrations of carvacrol was analyzed using a one-way analysis of variance (ANOVA) followed by Tukey’s test with a 95% confidence interval. An independent *t*-test was used to determine the significant differences in bacterial counts between treated and non-treated samples on individual days, while a two-way ANOVA was used to analyze the significant effect of different treatment methods on food matrices across the experimental period. The differences observed were considered to be statistically significant if the *p* value was <0.05. All statistical analyses were performed using IBM SPSS Statistics (version 29.0.0.0).

## Figures and Tables

**Table 1:** Host range of newly isolated coliphages.

*E. coli* Strains	Coliphage			
	SUT_E420	SUT_E520	SUT_E1520	SUT_E1620
ATC25922	-	-	-	+
K12	+	+	H	H
G106	+	+	+	(+)
G131	+	+	+	+
O157:H7	H	H	-	+
O113:H21	-	-	+	-
O111 (non-motile)	-	-	-	-
O130:H11	-	-	-	-
O15	-	(+)	-	+
O26:H11	+	-	-	-
O5 (non-motile)	-	-	-	-
O127:H6	-	-	-	+
O111	-	-	-	-
O119	-	-	-	(+)
O142:H6	-	-	-	+
O55:H6	-	-	-	-
O55:H7	(+)	(+)	-	(+)
*Salmonella* strains				
*S. Typhi*	-	-	-	+
*S. Enteritidis*	-	-	-	-
*S. Paratyphi*	-	-	-	+
*S. Hofit*	-	-	-	+
*S. Braenderup*	-	-	-	-
*S.e.s Newport*	-	(+)	-	-
*S.e.s Tallahassee*	-	-	-	-
*S.e.s Choleraesuis*	-	+	-	+
*S.e.s Anatum*	-	-	-	-

-: No lysis zone; +: Clear lysis zone; (+): Turbid lysis zone; *S.e.s*: *Salmonella enterica* serovar; H: Primary host strain.

**Table 2:** Sizes and dimensions of coliphages observed under TEM.

Phage	Head Length (nm)	Head Width (nm)	Tail Length (nm)	Tail Width (nm)
SUT_E420	73	73	175	11
SUT_E520	62	73	187	9
SUT_E1520	109	90	102	24
SUT_E1620	79	74	186	10

**Table 3:** Latent period and burst size calculated from one-step growth curve.

Phage	Latent Period (min)	Burst Size (PFU/CFU)
SUT_E420	30	350
SUT_E520	30	441
SUT_E1520	20	116
SUT_E1620	30	140

## Data Availability

Phage sequences generated from this study are published in the GenBank database and can be accessed with the accession numbers provided. Data reported in this study are not publicly available but can be provided upon request from the corresponding author.

## Supplementary Materials

The following supporting information can be downloaded at: https://www.mdpi.com/article/10.3390/antibiotics12111579/s1. Table S1: The efficiency of plating (EOP) of isolated coliphages against 13 *Escherichia* strains and five *Salmonella* strains. EOP ≥ 0.5, high efficiency; 0.5 > EOP ≥ 0.1, medium efficiency; EOP ≤ 0.1, low efficiency; H, primary host.
