# Supplementary material for: Control of Escherichia coli in Fresh-Cut Mixed Vegetables Using a Combination of Bacteriophage and Carvacrol
Source: Antibiotics (Basel). 2023 Oct 30;12(11):1579. doi: 10.3390/antibiotics12111579 (PMC10668671; doi:10.3390/antibiotics12111579)
Supplement: Supplementary file 1 [file antibiotics-12-01579-s001.zip › antibiotics-2662348-supplementary.pdf]

Supplementary Materials

Table S1: The efficiency of plating (EOP) of isolated coliphages against 13 *Escherichia* strains and five *Salmonella* strains. EOP ≥ 0.5, high efficiency; 0.5 > EOP ≥ 0.1, medium efficiency; EOP ≤ 0.1, low efficiency; H, primary host.

**Bacteria**	**SUT_E420**	**SUT_E520**	**SUT_E1520**	**SUT_E1620**
E. coli ATC25922	-	-	-	<0.001
E. coli K12	0.66	0.67	H	H
E. coli G106	0.74	0.73	0.44	<0.001
E. coli G131	0.69	0.92	0.91	0.58
E. coli O157:H7	H	H	-	0.25
E. coli O113:H21	-	-	0.03	-
E. coli O130:H11	-	-	-	-
E. coli O15	-	-	-	<0.001
E. coli O26:H11	<0.001	-	-	-
E. coli O127:*H6*	-	-	-	<0.001
E. coli O119	-	-	-	<0.001
E. coli O142:H6	-	-	-	<0.001
E. coli O55:H7	0.001	<0.001	-	<0.001
Salmonella Hofit	-	-	-	<0.001
Salmonella Typhi	-	-	-	0.26
Salmonella Paratyphi	-	-	-	<0.001
S.e.s Newport	-	<0.001	-	-
S.e.s Choleraesuis	-	<0.001	-	0.02

S.e.s: Salmonella enterica serovar
